# Duration of the common cold and similar continuous outcomes should be analyzed on the relative scale: a case study of two zinc lozenge trials

**DOI:** 10.1186/s12874-017-0356-y

**Published:** 2017-05-12

**Authors:** Harri Hemilä

**Affiliations:** Department of Public Health, POB 20 University of Helsinki, Tukholmankatu 8 B, FI-00014 Helsinki, Finland

**Keywords:** Data interpretation, Meta-analysis, Outcome assessment, Randomized controlled trial, Respiratory tract infections, Statistics, Zinc lozenges

## Abstract

**Background:**

The relative scale has been used for decades in analysing binary data in epidemiology. In contrast, there has been a long tradition of carrying out meta-analyses of continuous outcomes on the absolute, original measurement, scale. The biological rationale for using the relative scale in the analysis of binary outcomes is that it adjusts for baseline variations; however, similar baseline variations can occur in continuous outcomes and relative effect scale may therefore be often useful also for continuous outcomes. The aim of this study was to determine whether the relative scale is more consistent with empirical data on treating the common cold than the absolute scale.

**Methods:**

Individual patient data was available for 2 randomized trials on zinc lozenges for the treatment of the common cold. Mossad (Ann Intern Med 125:81–8, 1996) found 4.0 days and 43% reduction, and Petrus (Curr Ther Res 59:595–607, 1998) found 1.77 days and 25% reduction, in the duration of colds. In both trials, variance in the placebo group was significantly greater than in the zinc lozenge group. The effect estimates were applied to the common cold distributions of the placebo groups, and the resulting distributions were compared with the actual zinc lozenge group distributions.

**Results:**

When the absolute effect estimates, 4.0 and 1.77 days, were applied to the placebo group common cold distributions, negative and zero (i.e., impossible) cold durations were predicted, and the high level variance remained. In contrast, when the relative effect estimates, 43 and 25%, were applied, impossible common cold durations were not predicted in the placebo groups, and the cold distributions became similar to those of the zinc lozenge groups.

**Conclusions:**

For some continuous outcomes, such as the duration of illness and the duration of hospital stay, the relative scale leads to a more informative statistical analysis and more effective communication of the study findings. The transformation of continuous data to the relative scale is simple with a spreadsheet program, after which the relative scale data can be analysed using standard meta-analysis software. The option for the analysis of relative effects of continuous outcomes directly from the original data should be implemented in standard meta-analysis programs.

**Electronic supplementary material:**

The online version of this article (doi:10.1186/s12874-017-0356-y) contains supplementary material, which is available to authorized users.

## Background

In this study, the “absolute scale” indicates comparison on the scale of the original measurements such as days in the case of common cold duration. The “relative scale” indicates comparison with a placebo group level normalized to 1.0 or 100%.

The relative scale has been used for decades in analysing binary data in epidemiology. Relative risk (RR) allows the generalization of effects to different population groups, such as the 2-fold increase in total mortality and the 10-fold increase in lung cancer risk with smoking [1, 2]. Meta-analyses of binary outcomes on the relative scale have led to less heterogeneity than analyses on the absolute scale (risk difference) [3]. In addition, the relative scale cannot yield negative predicted values, whereas the absolute scale can.

In contrast, there has been a long tradition of carrying out meta-analyses of continuous outcomes on the absolute (original measurement) scale as the mean difference (MD), or using the standard deviation (SD) as the unit of the scale, which leads to the standardized mean difference (SMD) scale. Both of these approaches are available as options in popular meta-analysis software such as the RevMan program of the Cochrane collaboration [4].

The biological rationale for using the relative scale in the analysis of binary outcomes is that it adjusts for baseline variations. However, similar baseline variations can occur in continuous outcomes. For example, over 100 viruses cause the common cold, and the severity and duration of symptoms vary by virus. Since the distribution of viruses varies and different operational common cold definitions have been used in controlled trials, a substantial variation in the average untreated (placebo group) common cold duration is to be expected between trials. Since analysing the effect of treatment on the relative scale would partly adjust for such baseline variations between the placebo groups, the relative effect of a treatment on common cold duration might be more widely generalized than the absolute effect.

Friedrich et al. compared the absolute and relative scales in a series of 143 meta-analyses of continuous outcomes, and found less heterogeneity when the analysis was carried out on the relative scale [5]. They introduced the term Ratio of Means (RoM) to describe the calculation of the relative effect, and used a Taylor series-based approximation to calculate the SD for the RoM [5–7].

In our 2004 Cochrane review on vitamin C and the common cold, we used the relative scale, dividing the mean and SD of the cold durations of the study groups by the placebo group mean [8]. This approach is transparent and the SDs and the numbers of participants are apparent in the forest plots constructed with the standard programs. The effect estimate is identical with the RoM, yet the SD differs from that calculated by the Taylor series-based formula [5].

In epidemiology, the term RR is well established and the effects are often so large that the RR values are useful in communication. However, the treatment effects are often rather small and, instead of an RoM value, it may be more practical to consider treatment effects in percentages. Obviously, it is mathematically identical to state either that a treatment has an effect of RoM = 0.75 or that the treatment leads to a 25% reduction in the outcome. In this study, percentages are mainly used since they are familiar to most people.

If the relative scale is superior for some continuous outcomes compared with the absolute (i.e., MD) and the SMD scales, then the lack of the option of calculating the relative effect in popular meta-analysis packages would have led to suboptimal analysis of such outcomes. Although the calculation of the RoM estimates is simple on a spreadsheet, many meta-analysts do not consider approaches outside of the options provided by the standard programs. Furthermore, poorly selected measures of treatment effect can also hamper the communication of results to physicians and patients [5].

In considering the preference for the relative or the absolute scale, a question of fundamental importance is which of them is more consistent with the empirical data.

The approach of this study was to examine the predicted effects of zinc lozenges on the placebo group common cold durations by the absolute effect (in days) against the relative effect (in percentages). If the relative effect estimate is better, its application to the observed placebo group distribution would lead to a distribution of cold durations that is closer to the zinc lozenge group distribution. This kind of approach is informative only if the trial has demonstrated a significant difference between the treatment groups. This study analyses 2 trials that found benefit from zinc lozenges and for which individual patient data (IPD) were available [9, 10].

## Methods

### Selection of the zinc lozenge trials

No new literature search was done for this analysis. Several systematic searches of the literature on zinc lozenges and the common cold have been published [11–14] and the selection of the 2 trials for this analysis was based on those searches. The Mossad et al. [9] and the Petrus et al. [10] trials were selected because individual patient data (IPD) were available and because both of them found that zinc lozenges had a significant effect on common cold duration (Additional file 1). Significant benefit from zinc lozenges was reported in 2 other trials which published their results as survival curves. However, the follow-up was just one week in one of them and half of placebo patients were censored [15]. In another trial, the total number of patients was only 48 [16], half of the number of patients in both of the trials included [9, 10].

### Statistical methods

The Mossad [9] data set was no longer available and it was regenerated from the published survival curves as described previously [12] (Additional file 2). In the Mossad [9] study, there were 2 censored patients in the zinc lozenge group and 6 in the placebo group. In the analysis of the distributions of cold duration, the day of censoring was assumed to be the day of recovery. In the placebo group, 4 of the 6 censored patients were censored on day 15 or later. Thus, assuming that the censoring day was the day of recovery does not inflate the variance in the placebo group, but may bias it downwards. In the survival analyses, those patients were classified as censored. The Petrus [10] data set was kindly made available by Dr. Petrus; there were no censored patients in that study.

In this study, transformation to the percentage (relative) scale was done by dividing the means and SDs of the trial by the placebo group mean, and multiplying by 100% (Table 1). The zinc lozenge group level thus becomes the ratio of means (RoM) [5] and the difference between the zinc and placebo groups gives the effect of zinc lozenges in percentages directly. As a *t*-test, this approach is identical with the *t*-test of the original values (i.e., the MD scale), since the transformation is linear.

**Table 1:** Absolute and relative effects of zinc lozenges in the two randomized trials

	Mossad (1996) [9]		Petrus (1998) [10]	
Trial groups:	Zinc	Placebo	Zinc	Placebo
N:	49	50	52	49
Duration of colds				
Mean (days):	5.20	9.20	5.29	7.06
SD (days):	2.83	5.32	2.57	3.91
P, variance test to compare the SDs:	2*10^−5^		0.004	
Effect of zinc on the absolute scale (days): (95% CI):	−4.00 (−5.7 to −2.3)		−1.77 (−3.1 to −0.47)	
Quantiles of common cold duration (days)				
1st quartile:	3	5	3	4
2nd quartile:	5	8	5	6
3rd quartile:	7	14	7	8
90th percentile:	9	17	8	14
Transformation to the %-scale*:				
Mean (% of placebo group mean):	56.6%	100%	74.9%	100%
SD (% of placebo group mean):	30.7%	57.8%	36.4%	55.3%
Effect of zinc on the relative scale: (95% CI):	−43.4% (−62% to −24.9%)		−25.1% (−44% to −6.7%)	

* Transformation to the %-scale indicates that the means and SDs of the Mossad [9] study were divided by 9.20 days, and the Petrus [10] study by 7.06 days. By this transformation the value of the zinc lozenge group mean becomes the RoM and the difference between the zinc and placebo groups gives the effect of zinc lozenges directly in percentages. Since this transformation is linear, the relative scale leads to a *t*-score identical with that obtained on the original values (i.e., on the absolute scale; the MD scale)

Other approaches to analyze the relative effect of zinc lozenges are shown in Additional file 3. The second way to calculate the 95% CI and the *P*-value was done by first log-transforming the cold durations, which makes the variances of the zinc and placebo groups equal (*P* > 0.4; variance test for both trials), but increases skewness in Mossad’s zinc group, thereafter calculating the *t*-test on the log scale, and finally back-transforming to the relative effect estimate and its 95% CI. The third way to calculate the 95% CIs was by the Taylor series-based formula [5]. The fourth way to calculate the 95% CI was by the Fieller method allowing unequal variances [17]. Finally, the 95% CI was calculated by bootstrap. This was done on both the normalized scale so that the placebo group mean was set at 100%, i.e., sampling [mean(Zn) – mean(Placebo = 100%)], and on the ratio of the means, i.e., sampling [mean(Zn)/mean(Placebo)].

The R program package [18] was used in the statistical analyses. The similarity of the variance in the zinc and placebo groups was tested by the var.test procedure. The *t*-test was calculated by the t.test procedure (var.equal = False/True). The Fisher-Pitman permutation test was calculated by the oneway_test procedure (distribution = exact) of the coin package. The Fieller 95% CIs were calculated with the t.test.ratio procedure of the mratios package. The bootstrap 95% CIs were calculated by the boot and boot.ci procedures of the boot package (type = BCa). Cox regression models were calculated by the coxph procedure, the logrank test by the survdiff(rho = 0) procedure and the Kaplan-Meier curves were drawn using the survfit procedure of the survival package. The calculations are described in Additional file 4.

## Results

This comparison of the absolute scale against the relative scale in the analysis of the treatment effects on common cold duration focused on 2 randomized trials; the zinc gluconate lozenge trial by Mossad et al. [9] and the zinc acetate lozenge trial by Petrus et al. [10]. Both trials had close to 100 patients divided equally into their zinc and placebo groups (Table 1; Additional file 1).

### The Mossad [9] trial

**Fig. 1:**
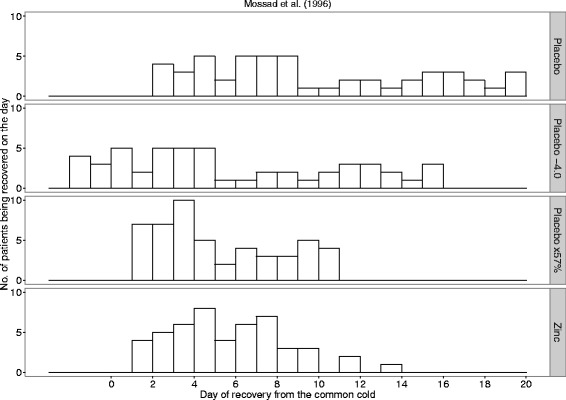
The observed days of recovery and the transformed placebo group days of recovery in the Mossad [9] study. The first row shows the observed number of placebo patients who recovered on the day shown on the horizontal axis. The bottom row shows the observed number of zinc lozenge patients who recovered on the given day. The SDs of the placebo and zinc lozenge groups are significantly different with *P* = 2*10^−5^. The second row shows the transformation in which each placebo group common cold is assumed to become 4.0 days shorter because of the effect of the zinc lozenges. The third row shows the transformation in which each placebo group common cold is assumed to become 43% shorter because of the zinc lozenges. In the Mossad study, 2 patients were censored in the zinc group on days 9 and 11; and 6 patients were censored in the placebo group on days 7, 15, 16, and 19. The day of censoring was assumed to be the day of recovery in this figure

Figure 1 shows the number of common cold patients who recovered on each day of the follow-up in the Mossad [9] trial. The placebo group is on the top row and the zinc lozenge group on the bottom row of the figure. Both distributions appear closer to the uniform distribution than to the normal distribution. Furthermore, the SD of the placebo group is significantly greater than the SD of the zinc group (Table 1).

On the absolute scale, zinc lozenges shortened the duration of colds by 4.0 days. Table 1 shows the transformation of the common cold duration to the relative scale so that the placebo group is normalized to 100%. The zinc group level becomes the RoM = 0.566 and the difference between the zinc and placebo groups directly indicates a 43.4% reduction in common cold duration with the zinc lozenges. Since this transformation is linear, the *t*-tests for the 2 approaches lead to identical *t*-scores and *P*-values. The relevant question about the preference for the 2 effect estimates, 4.0 days vs. 43%, is the divergence in their implications.

If the 4-day reduction in common cold duration is assumed to be a uniform expected effect over Mossad’s patients then the 4-day estimate should be valid for both short and long colds. This implies that the 4-day reduction in cold duration with zinc lozenges corresponds to the translocation of the common cold distribution of the placebo group towards the left by 4.0 days (2nd row in Fig. 1). The uniform shortening of colds by 4.0 days predicts that zinc treatment would lead to 12 patients with a negative or zero cold duration, which are impossible. Such a transformation also retains the high variance of the placebo group compared with the actual zinc lozenge group.

If the 43% reduction in common cold duration is assumed to be a uniform relative effect over Mossad’s patients, this indicates that multiplication of the cold durations of the placebo group by 0.57 should lead to a distribution similar to the distribution of the zinc group (3rd and 4th rows in Fig. 1). The 43% shortening of colds in the placebo group does not predict impossible, i.e., negative or zero, cold durations and the SD of this predicted distribution is consistent with the SD of the actual zinc lozenge group (variance test *P* = 0.7).

The absolute and relative effects can also be compared on the basis of the quartiles of the cold distributions in the treatment groups (Table 1). The ratios for the 1st, 2nd and 3rd quartiles of the zinc and placebo groups indicate reductions in common cold duration by 40, 38 and 50% respectively with zinc lozenges, which are all close to the overall 43% estimate. In contrast, the absolute differences in the quartiles of the 2 groups are 2, 3 and 7 days, which have a much greater variation around the absolute effect estimate of 4.0 days. For the 90th percentiles of cold duration, the bootstrap 95% CI for the absolute difference between the zinc and placebo groups is from −11 to −5.6 days, which does not include the overall −4.0 day estimate. In contrast, the bootstrap of the ratio of the 90th percentiles gives a 95% CI from −58 to −31%, which is consistent with the overall estimate of −43%.

**Fig. 2:**
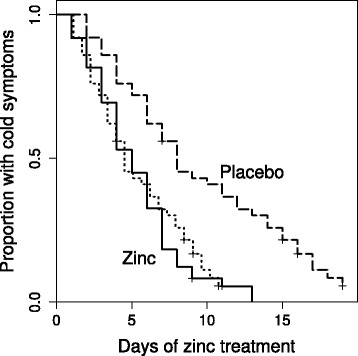
Survival curves for the treatment groups and for the 43% shortened placebo group cold durations for the Mossad [9] study. The solid curve indicates the zinc lozenge group, the dashed curve on the right hand side indicates the placebo group, and the dotted curve overlapping the zinc lozenge curve indicates the recovery curve obtained by shortening all the placebo group common cold durations by 43%. The difference between the placebo group and the zinc lozenge group is significant, *P* = 10^−5^ on the logrank test, whereas there is no difference between the zinc lozenge group and the 43% shortened placebo group common cold durations (*P* = 0.8). The 4.0-day shortened placebo group colds (2nd row in Fig. 1) would lead to a survival curve starting to decline before day 0 and crossing the zinc group curve. To keep this figure less confusing that curve is not shown. Cox regression model indicates that zinc lozenges increased the rate of recovery by RR = 2.7 (95% CI 1.7 to 4.4)

Survival analysis is a further informative way to examine the time of recovery. The Kaplan-Meier plots for the zinc group and the 43% shortened placebo group cold durations are closely overlapping (Fig. 2). Cox regression gives an estimate of RR = 2.7 for the effect of zinc lozenges.

### The Petrus [10] trial

**Fig. 3:**
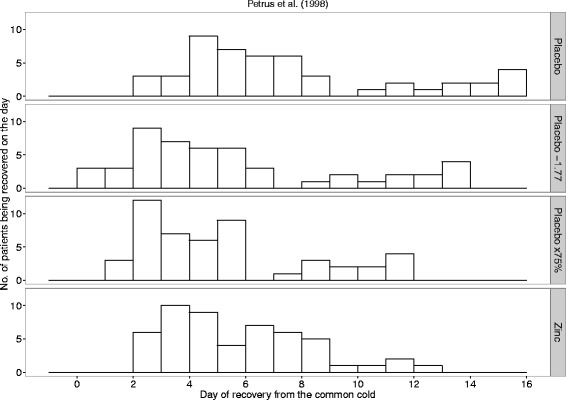
The observed days of recovery and the transformed placebo group days of recovery in the Petrus [10] study. The first row shows the observed number of placebo patients who recovered on the day shown on the horizontal axis. The bottom row shows the observed number of zinc lozenge patients who recovered on the given day. The SDs of the placebo and zinc lozenge groups are significantly different with *P* = 0.004. The second row shows the transformation in which each placebo group common cold is assumed to become 1.77 days shorter because of the effect of the zinc lozenges. The third row shows the transformation in which each placebo group common cold is assumed to become 25% shorter because of the zinc lozenges

Figure 3 shows the number of patients recovering on each day in the Petrus [10] trial. Here too the distributions of colds in the placebo and zinc groups appear close to the uniform distribution and the SD is significantly greater in the placebo group than the zinc group (Table 1). Zinc lozenges shortened the duration of colds by 1.77 days on the absolute scale and by 25% on the relative scale (Table 1).

The 1.77-day uniform shortening of colds in the placebo group predicts that 3 colds will last just for 0 days (2nd row in Fig. 3) and retains the high variance compared with the actual zinc lozenge group. In contrast, the 25% shortening of colds in the placebo group leads to a cold distribution similar to the zinc lozenge group (3rd and 4th rows of Fig. 3), and to a SD consistent with that of the zinc group (variance test *P* = 0.4).

The quartiles of duration in the Petrus [10] trial are not informative for the comparison of the absolute and relative scales. However, the difference between the zinc and placebo groups at the 90th percentile level of duration, −43% and −6 days, is more consistent with the relative effect estimate of −25% compared with the absolute effect estimate of −1.77 days (Table 1). For the 90th percentiles, the bootstrap 95% CI for the difference between the groups is from −8 to −4.5 days, which is far from the overall estimate of −1.77 days. In contrast, the bootstrap of the ratio of the 90th percentiles gives a 95% CI from −53 to −33%, which is much closer to the overall estimate of −25%.

**Fig. 4:**
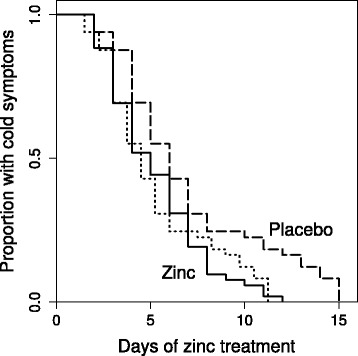
Survival curves for the treatment groups and for the 25% shortened placebo group cold durations for the Petrus [10] study. The solid curve indicates the zinc lozenge group, the dashed curve on the right hand side indicates the placebo group, and the dotted curve overlapping the zinc lozenge curve indicates the recovery curve obtained by shortening all the placebo group common cold durations by 25%. The difference between the placebo group and the zinc lozenge group is significant, *P* = 0.006 on the logrank test, whereas there is no difference between the zinc lozenge group and the 25% shortened placebo group common cold durations (*P* = 0.9). Cox regression model indicates that zinc lozenges increased the rate of recovery by RR = 1.77 (95% CI 1.16 to 2.7)

The Kaplan-Meier plots for the zinc group and the 25% shortened placebo group colds overlap closely (Fig. 4). Cox regression gives an estimate of RR = 1.77 for the rate of recovery in the zinc lozenge group compared with the placebo group.

### Comparison of methods available to calculate the *P*-values and the 95% CIs for relative effects

Given that the relative scale appears better in the analysis of common cold duration, different approaches to calculate the *P*-values and the 95% CIs for relative effects were compared (Additional file 3). Since both trials had significant differences in the variances between the zinc lozenge and placebo groups, the *t*-test was calculated assuming equal variance and allowing unequal variance, but the difference in the *P*-values was minor. The nonparametric Fisher-Pitman permutation test and the logrank test gave *P*-values essentially identical with those by the *t*-tests. For both trials, the *t*-test of log-transformed cold durations yielded conservative *P*-values compared with the *P*-values calculated with the permutation and logrank tests. The 95% CIs calculated with the relative scale approach used in this study were more conservative than the 95% CIs calculated by the Taylor series approach [5] and by the Fieller approach [17].

## Discussion

If a treatment consistently has no effect at all, the scale of the meta-analysis probably matters little. However, if a treatment does have an effect, the scale may substantially influence the interpretation of the findings and the communication about them. Therefore this analysis selected 2 trials that found a significant benefit from zinc lozenges and for which IPD was available. While the goal of this study was not to estimate the overall effect of zinc lozenges on common cold duration, the findings in these 2 trials are consistent with the relative effects (i.e., the % decrease) found in 4 other trials on zinc lozenges [12, 19].

When the effect of zinc lozenges on cold duration was estimated on the absolute scale, i.e., as the number of days by which the colds became shorter, applying the effect estimates to the placebo group cold distribution yielded impossible, negative or zero, predicted durations for colds. In addition, the distributions remained wide compared with the distributions in the actual zinc lozenge groups. In contrast, when the relative effect estimate was applied to the placebo group cold distributions, no impossible common cold durations were predicted, and the cold distributions became similar to those of the zinc lozenge groups.

Calculation of the relative effect on common cold duration is conceptually consistent with the RR calculated by Cox regression. Nevertheless, the estimates should not be expected to be reciprocals since the RR depends on the shapes of the curves, which can differ substantially even though the mean durations remain invariant. Compared with the *t*-test, survival analysis is a superior way to analyse the recovery rate at the level of individual studies (Figs. 2 and 4), since it allows visual inspection and formal analysis of time-dependent changes in the treatment effect, and is not hampered by censored data or outlier patients who have exceptionally long colds. In contrast, outliers lead to inflation of the SD estimates in the *t*-test and may decrease the power to demonstrate an effect. However, since only the means and SDs of the study groups are usually published, survival analysis is rarely an option in the meta-analysis of disease duration. Therefore, in the meta-analyses of time data the usual question is about whether the absolute (i.e., MD) scale or the relative scale is more reasonable for the *t*-test.

The selection of scale is important in 2 respects. First, the scale is important when several studies are pooled in a meta-analysis, since one scale can lead to less heterogeneity than another, indicating better capture of the effect by the former scale [3]. Second, the scale influences the communication about the findings of single trials and of meta-analyses.

There has been substantial variation in the mean duration of untreated (placebo group) colds between trials. In our Cochrane review on vitamin C and the common cold, the shortest mean duration of placebo group colds in trials with children was 2.8 days, whereas the longest was 14 days [20, 21]. Because the untreated colds differ so greatly, a 1-day effect has a very different meaning in those two trials, even though the nominal value of the effect is the same. Furthermore, with an effective treatment, the 14-day colds might be shortened by a week, whereas such an effect does not exist on the 2.8-day scale of the former trial. Thus, the absolute scales of those two trials are incompatible. Therefore, we have used the relative scale in our Cochrane review on vitamin C and the common cold since 2004 [8, 20]. There has also been a substantial variation in cold durations in the placebo groups of 11 zinc lozenge studies, from 5 to 10 days [12].

There is evidence from studies on vitamin C and the common cold that heterogeneity in treatment effect may be lower on the relative scale. In 14 trials on vitamin C for children, the relative scale (i.e., the %-scale) pooling led to I^2^ = 27% (*P* = 0.17) for the heterogeneity in the treatment effects, whereas heterogeneity in the days-scale was I^2^ = 46% (*P* = 0.03). Less heterogeneity may lead to stronger evidence of treatment effect and, as expected, the pooled treatment effect on the relative scale was stronger (Z = 4.04) than on the absolute scale (Z = 3.11) [21]. An earlier meta-analysis of vitamin C and the common cold studies also pointed out that the relative scale led to a stronger evidence of treatment effect (*P* = 0.001) than the absolute scale (*P* = 0.01) [22]. These differences indicate that the relative scale may capture the effects of treatments on common cold duration better.

The selection of scale is also important in the communication of the findings. In the placebo groups of the included trials, the duration of the common colds ranged from 2 to 19 days in the Mossad [9] trial, and from 2 to 15 days in the Petrus [10] trial. Such a great variation in the durations of untreated colds should be taken into account in communication. The 43 and 25% effects are applicable over the whole range of the untreated cold durations. In contrast, claims that zinc shortens the duration of colds by 4.0 or 1.77 days according to the two trials analyzed (Table 1), or by 1.65 or 1.03 days according to two meta-analyses [13, 14], have a very different meaning depending on whether the assumed untreated cold episode might last for 2 days or 2 weeks. If only one type of estimate is used in the communication, the relative effect appears to be much more informative since it is applicable to the entire range of potential episode durations. Nevertheless, both measures may be shown in parallel.

The SMD scale is a third approach that has been used to estimate the magnitude of treatment effect on continuous outcomes [4, 5]. This normalizes the observations so that one unit on the scale corresponds to one unit of SD in each trial included in the meta-analysis. Such a scale is confusing for an ordinary reader. For example, the 2011 Cochrane review on zinc and the common cold stated in its abstract that the “intake of zinc is associated with a significant reduction in the duration (standardised mean difference (SMD) -0.97)” [23]. Reporting should always show the unit of measurement, and this sentence should have been written more accurately as: “zinc shortened the duration of colds by 0.97 SD units”. However, such accurate reporting would have revealed the main problem of the SMD scale: what does the SD unit mean in practical terms. Most physicians and patients can consider whether 42 or 25% is a small or a large effect, but few of them can form their own opinion about whether an effect of 0.97 SD units is small or large. In this respect, the relative scale is far superior in the communication of findings to physicians and patients, since they have long-term familiarity with the percentage effects. The difficulty of communicating the SMD findings to patients has been pointed out [5, 24].

The SMD scale is extensively used. However, its usage does not seem to originate from biological or statistical considerations, but from the fact that, along with the MD scale, it is the only option that is available in the popular statistical software for meta-analysis, such as the RevMan of Cochrane Collaboration [4]. Thus, the wide availability of the SMD option guides researchers to use it without them considering the biological issues properly, or the difficulties in communicating the findings on that scale [5, 24].

Based on 143 meta-analyses on continuous outcomes, Friedrich et al. concluded that there was less heterogeneity in the meta-analyses when they were carried out on the relative scale [5]. Although their findings support the preference for the relative scale, they did not consider the diversity of the kinds of outcomes that are measured by continuous outcomes. It seems evident that the relative scale is superior for some outcomes such as the duration of the common cold and other diseases, whereas the absolute scale may be superior for some other outcomes. Therefore diverse continuous outcomes should not be combined into a uniform mass, so that all of them are calculated on the relative scale and compared with all of them on the absolute scale.

Essential requirements for using the relative scale in a meta-analysis appear to be that the measurements can be transformed so that the control group means are 100%, and that there is a relevant and unambiguous target level of 0%. However, there are many types of continuous outcomes which do not have a relative scale that is relevant. For example, since blood pressure does not have either a reasonable 100% level over various trials, or a reasonable 0% target level, pooling studies and reporting effects on blood pressure on the percentage scale might lead to confusions. The mmHg scale (absolute scale) stratified by the pre-treatment mmHg level may be preferable. Another example where percentage scale would lead to confusions is measuring body temperature. If a patient has fever, it is more informative to describe how many degrees the fever was reduced, rather than describing the relative decrease in body temperature on the Kelvin scale.

The duration of the common cold was used as a model of continuous outcomes. The common cold is by itself a clinically relevant topic as reflected, for example, by the existence of 18 Cochrane reviews in which the title includes the term [25]. Furthermore, it seems evident that the findings of this study apply to many other continuous outcomes that measure time, such as the duration of other diseases, and the duration of hospital stay and intensive care unit stay, etc.

In this study, the transformation to the relative scale was done by dividing the mean and SD values by the placebo group mean value, which transforms the zinc group mean level to the RoM and keeps the ratios of SDs and means identical with their ratios on the absolute scale. This approach is transparent but more conservative than the Taylor series approach [5] and the Fieller approach [17], see Additional file 3. These approaches should be compared to find out which is the most useful in meta-analyses.

## Conclusions

The choice between the absolute scale and the relative scale should be determined on the basis of biological reasoning and on the basis of testing which scale leads to less heterogeneity. The relative scale option should be made available for the standard meta-analysis software, but meanwhile the transformation to the relative scale can be easily calculated with spreadsheet programs, and the transformed data can be analyzed with standard meta-analysis software.

## Additional files

Additional file 1: Description of the two trials. (PDF 66 kb)

Additional file 2: Data analyzed in the study. (PDF 127 kb)

Additional file 3: Comparison of methods available to calculate the *P*-values and the 95% CIs for relative effects. (PDF 65 kb)

Additional file 4: Description of the calculations. (PDF 98 kb)

## Abbreviations

IPD: Individual patient data
MD: Mean difference
RoM: Ratio of means
RR: Relative risk
SD: Standard deviation
SMD: Standardized mean difference

## References

[1] Doll R, Peto R (1976). Mortality in relation to smoking: 20 years’ observations on male British doctors. Br Med J.

[2] Lee PN, Forey BA, Coombs KJ (2012). Systematic review with meta-analysis of the epidemiological evidence in the 1900s relating smoking to lung cancer. BMC Cancer.

[3] Deeks JJ (2002). Issues in the selection of a summary statistic for meta-analysis of clinical trials with binary outcomes. Stat Med.

[4] Review Manager (RevMan) [Computer program]. Version 5.3. Copenhagen: The Nordic Cochrane Centre, The Cochrane Collaboration. http://community.cochrane.org/tools/review-production-tools/revman-5. Accessed 9 May 2017.

[5] Friedrich JO, Adhikari NK, Beyene J (2011). Ratio of means for analyzing continuous outcomes in meta-analysis performed as well as mean difference methods. J Clin Epidemiol.

[6] Friedrich JO, Adhikari NK, Beyene J (2008). The ratio of means method as an alternative to mean differences for analyzing continuous outcome variables in meta-analysis: a simulation study. BMC Med Res Methodol.

[7] Friedrich JO, Adhikari NK, Beyene J (2012). Ratio of geometric means to analyze continuous outcomes in meta-analysis: comparison to mean differences and ratio of arithmetic means using empiric data and simulation. Stat Med.

[8] Douglas RM, Hemilä H, D'Souza R, Chalker EB, Treacy B (2004). Vitamin C for preventing and treating the common cold. Cochrane Database Syst Rev.

[9] Mossad SB, Macknin ML, Medendorp SV, Mason P (1996). Zinc gluconate lozenges for treating the common cold: a randomized, double-blind, placebo-controlled study. Ann Intern Med.

[10] Petrus EJ, Lawson KA, Bucci LR, Blum K (1998). Randomized, double-masked, placebo-controlled clinical study of the effectiveness of zinc acetate lozenges on common cold symptoms in allergy-tested subjects. Curr Ther Res.

[11] Eby GA (2010). Zinc lozenges as cure for the common cold - a review and hypothesis. Med Hypotheses.

[12] Hemilä H (2011). Zinc lozenges may shorten the duration of colds: a systematic review. Open Respir Med J.

[13] Science M, Johnstone J, Roth DE, Guyatt G, Loeb M (2012). Zinc for the treatment of the common cold: a systematic review and meta-analysis of randomized controlled trials. CMAJ.

[14] Singh M, Das RR (2013). Zinc for the common cold. Cochrane Database Syst Rev.

[15] Eby GA, Davis DR, Halcomb WW (1984). Reduction in duration of common cold by zinc gluconate lozenges in a double-blind study. Antimicrob Agents Chemother.

[16] Prasad AS, Fitzgerald JT, Bao B, Beck FW, Chandrasekar PH (2000). Duration of symptoms and plasma cytokine levels in patients with the common cold treated with zinc acetate: a randomized, double-blind, placebo-controlled trial. Ann Intern Med.

[17] Tamhane AC, Logan BR (2004). Finding the maximum safe dose level for heteroscedastic data. J Biopharm Stat.

[18] R Core Team (2016) R Project for Statistical Computing. https://www.r-project.org. Accessed 14 Apr 2017.

[19] Hemilä H. Zinc lozenges and the common cold: a meta-analysis comparing zinc acetate and zinc gluconate, and the role of zinc dosage. JRSM Open. 2017;8(5):2054270417694291. https://doi.org/10.1177/2054270417694291. 10.1177/2054270417694291 PMC5418896 28515951

[20] Hemilä H, Chalker E (2013). Vitamin C for preventing and treating the common cold. Cochrane Database Syst Rev.

[21] Hemilä H (2016). Many continuous variables such as the duration of the common cold should be analyzed using the relative scale. J Clin Epidemiol.

[22] Hemilä H, Herman ZS (1995). Vitamin C and the common cold: a retrospective analysis of Chalmers’ review. J Am Coll Nutr.

[23] Singh M, Das RR (2011). Zinc for the common cold. Cochrane Database Syst Rev.

[24] Johnston BC, Alonso-Coello P, Friedrich JO, Mustafa RA, Tikkinen KA, Neumann I (2016). Do clinicians understand the size of treatment effects? A randomized survey across 8 countries. CMAJ.

[25] Cochrane Library. http://www.cochranelibrary.com/. Accessed 14 Apr 2017.

