# Supplementary material for: Duration of the common cold and similar continuous outcomes should be analyzed on the relative scale: a case study of two zinc lozenge trials
Source: BMC Med Res Methodol. 2017 May 12;17:82. doi: 10.1186/s12874-017-0356-y (PMC5427521; doi:10.1186/s12874-017-0356-y)
Supplement: Supplementary file 1 — Description of the two trials. (PDF 66 kb) [file 12874_2017_356_MOESM1_ESM.pdf]

Duration of the common cold and similar continuous outcomes should be analyzed on the relative scale: a case study of two zinc lozenge trials

Additional File 1: **Description of the two trials**

This is additional material to a paper by Hemilä (2017).
https://bmcmedresmethodol.biomedcentral.com

version April 14, 2017

Harri Hemilä
Department of Public Health,
University of Helsinki,
Helsinki, FIN-00014 Finland
harri.hemila@helsinki.fi
http://www.mv.helsinki.fi/home/hemila/
http://www.mv.helsinki.fi/home/hemila/Zinc.htm

**Mossad (1996) [9]**	http://dx.doi.org/10.7326/0003-4819-125-2-199607150-00001<br>http://www.annals.org/content/125/2/81<br>https://www.ncbi.nlm.nih.gov/pubmed/8678384
Methods	Randomized, placebo-controlled, double-blind trial
Randomization	“A statistical consultant prepared a computer-generated randomization code and the packages of medication. The packages were identical in appearance except for the randomization numbers. The study medication was distributed by the study nurse, who was masked to treatment assignments” (p. 82).
Allocation concealment	Participants and personnel did not know to which group the participants were allocated.
Blinding of participants and personnel	“Double-blind” (p. 81). “The study medication was distributed by the study nurse, who was masked to treatment assignments” (p. 82).
Blinding of outcome assessment	“[Blinded] patients were asked to complete a daily log documenting the severity of symptoms ” (p. 83).
Losses to follow-up	“One patient in the zinc group withdrew from the study on the first day because she could not tolerate the lozenges” (p. 83).
Participants	Included in the analysis: 49 Zn and 50 placebo participants.<br>19 M 80 F, mean age 37 yr (range 21 to 69 yr).<br>Participants were recruited from among the Cleveland Clinic staff through announcements in internal clinic publications<br>and by word of mouth.<br>Exclusions: pregnancy, a known immune deficiency.
Common cold definition	“Patients must have had ≥2 of the 10 following symptoms: cough, headache, hoarseness, muscle ache, nasal drainage, nasal congestion, scratchy throat, sore throat, sneezing, or an oral temperature >37.7°C” (p. 82).
Delay between cold onset and treatment initiation	Inclusion required that the cold had lasted for ≤24 hr (p. 82).
Outcome definition	“Cold resolution was defined as<br>resolution of all symptoms (a total symptom score of 0) or<br>resolution of all but one mild symptom (a total symptom score of 1)” (p. 83).
Intervention	Zn gluconate glycine: one lozenge contained 13.3 mg Zn<br>Placebo lozenges contained Ca lactate.<br>Participants were instructed to dissolve 1 lozenge in their mouth every 2 hr while awake.<br>The reported mean number of lozenges used per day in the Zn group was 6 (p. 84).
Daily Zn dose	80 mg/d = 6/d × 13.3 mg
Mean and SD of the common cold duration	Mossad (1996) reported (p. 84):<br>Zn group: **median** time to resolution of cold symptoms: 4.4 d<br>Placebo group: **median** time to resolution of cold symptoms: 7.6 d<br>Mossad published the survival curves for the zinc and placebo groups, which were measured in [12]. The recovery days were recalculated for this study (Additional file 2).<br>Zn group: mean duration colds: **5.204 d (SD 2.828)**<br>Placebo group: mean duration of cold: **9.200 d (SD 5.318).**

**Petrus (1998) [10]**	http://dx.doi.org/10.1016/S0011-393X(98)85058-3<br><br>http://www.currenttherapeuticres.com/article/S0011-393X%2898%2985058-3/abstract
Methods	Randomized, placebo-controlled, double-blind trial
Randomization	"The bottles of the zinc lozenges and placebo were sent by the manufacturer and each bottle was identical except a sequential number. At registration, after qualifying for the study each patient was given a bottle of 180 lozenges. At the conclusion of the study, when the diaries were assembled, the code for the bottles was sent by the manufacturer, and the patients were placed in the zinc or placebo category. Then the results were tabulated and the statistical analysis was undertaken" (Edward Petrus 24 March 2016).
Allocation concealment	Patients and personnel did not know to which group the patients were allocated.
Blinding of patients and personnel	"Double-masked" (p. 595). Participants and personnel were blinded.
Blinding of outcome assessment	Blinded "subjects were also informed that they were required to rate and record their symptoms in a diary " (p. 598). "Subjects recorded their symptoms every day until their symptoms ceased (p. 598).
Losses to follow-up	1 patient was lost to follow-up.
Patients	Included in the analysis: 52 Zn and 49 placebo patients.<br>47 M 54 F, mean age 26 yr (range 18 to 54 yr).<br>Participants were recruited from the campus of the University of Texas through posted announcements.<br>Exclusions: serious illnesses, organ transplants, disability.
Common cold definition	"subjects had to have ≥2 common cold symptoms (nasal drainage, nasal congestion, cough, fever, myalgia, headache, sore throat, scratchy throat, hoarseness, sneezing, or malaise) " (p. 598).
Delay between cold onset and treatment initiation	"97 of the 101 subjects started using zinc lozenges on the first day of enrollment in the study (4 started on day 2 of enrollment), but the dataset doesn't contain any information on the length of time between onset of symptoms and start of zinc therapy" (Kenneth Lawson, email 11 Dec 2014).
Outcome definition	Duration of the longest-lasting common cold symptom.
Intervention	Zn acetate: one lozenge contained 9 mg Zn (p. 597).<br>Placebo lozenges contained sucrose octaacetate.<br>Patients were instructed to use 1 lozenge every 1½ hour while awake during day 0, then 1 lozenge every 2 hour while awake on following days.<br>"averaged 9.9 lozenges per subject per day as long as symptoms persisted" (p. 599).
Daily Zn dose from the lozenges	89 mg/d = 9.9/d × 9 mg
Mean and SD of the common cold duration	Calculated from the data set (the same as reported in 1998):<br>Zn group: mean duration colds: **5.288 days (SD 2.569)**<br>Placebo group: mean duration of cold: **7.061 days (SD 3.907).**
