# Supplementary material for: Duration of the common cold and similar continuous outcomes should be analyzed on the relative scale: a case study of two zinc lozenge trials
Source: BMC Med Res Methodol. 2017 May 12;17:82. doi: 10.1186/s12874-017-0356-y (PMC5427521; doi:10.1186/s12874-017-0356-y)
Supplement: Supplementary file 2 — Data analyzed in the study. (PDF 127 kb) [file 12874_2017_356_MOESM2_ESM.pdf]

Duration of the common cold and similar continuous outcomes should be analyzed on the relative scale: a case study of two zinc lozenge trials

Additional File 2: **Data analyzed in the study**

This is additional material to a paper by Hemilä (2017).
https://bmcmedresmethodol.biomedcentral.com

version April 17, 2017

Harri Hemilä
Department of Public Health,
University of Helsinki,
Helsinki, FIN-00014 Finland
harri.hemila@helsinki.fi
http://www.mv.helsinki.fi/home/hemila/
http://www.mv.helsinki.fi/home/hemila/Zinc.htm

Page

2 The Mossad (1996) study imputations

4 The Mossad (1996) data set

5 The Prasad (1998) data set

The survival curve (recovery from colds curve) published by Mossad (1996) [9] was measured and transformed to participants being cured and censored in Hemilä (2011) [12]. Here the measurements were reanalyzed leading to minor changes compared with the previous analysis.

The zinc group of Mossad (1996) trial:

Mossad 1996

Zn (n=49)

The bold No:s are reported in Mossad's figure

**Day**	pixels measured 100%= 2274	To Fig scale (0 to 100%)	To persons	Based on censoring, (1 participant censored on 2 days) rescaling on the days 9th		11th		To person integers Remain	**Cured per day**	**Censored**	**Day**
**0**	213	100	49					**49**	0		**0**
**1**	384	91.7	44.93					45	4		**1**
**2**	589	81.76	40.06					40	5		**2**
**3**	850	69.09	33.86					**34**	6		**3**
**4**	1180	53.08	26.01					26	8		**4**
**5**	1345	45.08	22.09					22	4		**5**
**6**	1598	32.8	16.07					**16**	6		**6**
**7**	1893	18.49	9.06					9	7		**7**
**8**	2018	12.42	6.09					6	3		**8**
**9**	2102	8.35	4.09	> x3/4 >	3.07			**3**	2	1	**9**
**10**	2102	8.35	4.1		3.07			**3**	0		**10**
**11**	2168	5.14	2.5		1.89	> x1/2 >	0.95	**1**	1	1	**11**
**12**	2168	5.14	2.5		1.9		0.95	**1**	0		**12**
**13**	2274	0	0.0					**0**	1		**13**
								**n =**	**47**	**2**	

Mossad reported:

2 Zn participants dropped out after 7 to 16 days
The days were not reported and 9 and 11 days
are inferred from the Kaplan-Meier curves

The placebo group of Mossad (1996) trial:

Placebo (n=50)

**Day**	pixels measured	To Fig scale (0 to 100%)	To persons	Based on censoring, (1-2 participants censored on 3 days) rescaling on the days	7th		15th		16th	**The bold No:s are reported in Mossad's figure**<br>To person Integers Remain	**Cured per day**	**Censored**	**Day**
**0**	213	100	50							**50**	0		**0**
**1**	213	100	50							50	0		**1**
**2**	382	91.8	45.90							46	4		**2**
**3**	502	85.98	42.99							**43**	3		**3**
**4**	710	75.89	37.94							38	5		**4**
**5**	792	71.91	35.95							36	2		**5**
**6**	996	62.01	31.00							**31**	5		**6**
**7**	1116	56.19	28.09	> x26/28 >	26.09					26	3	**2**	**7**
**8**	1334	45.61	22.8		21.18					21	5		**8**
**9**	1379	43.43	21.7		20.16					**20**	1		**9**
**10**	1414	41.73	20.9		19.37					19	1		**10**
**11**	1511	37.02	18.5		17.19					17	2		**11**
**12**	1603	32.56	16.3		15.1					**15**	2		**12**
**13**	1644	30.57	15.3		14.2					14	1		**13**
**14**	1732	26.3	13.1		12.2					12	2		**14**
**15**	1835	21.3	10.7		9.9	> x9/10 >	8.90			**9**	2	1	**15**
**16**	1938	16.3	8.2		7.6		6.81	> x6/7 >	5.84	6	2	**1**	**16**
**17**	2050	10.87	5.4		5.0		4.5		3.89	4	2		**17**
**18**	2101	8.39	4.2		3.9		3.5		3.01	3	1		**18**
**19**										**0**	1	**2**	**19**
										**n =**	44	**6**	

Mossad reported:

4 placebo participants dropped out after 7 to 16 days

The days were not reported and 7, 15 and 16 days are inferred from the Kaplan-Meier curves

2 had censored data on the 19th day and 1 was cured on the 19th day

The Mossad (1996) data set

Total Observations in Table: 99

Mossad$Days	Mossad$Zinc 0	1
1	0	4
2	4	5
3	3	6
4	5	8
5	2	4
6	5	6
7	5	7
8	5	3
9	1	3
10	1	0
11	2	2
12	2	0
13	1	1
14	2	0
15	3	0
16	3	0
17	2	0
18	1	0
19	3	0
Column Total	50	49

The Petrus (1998) data set

Total Observations in Table: 101

	Petrus$Zinc	
**Petrus$Days**	**0**	**1**
2	3	6
3	3	10
4	9	9
5	7	4
6	6	7
7	6	6
8	3	5
9	0	1
10	1	1
11	2	2
12	1	1
13	2	0
14	2	0
15	4	0
Column Total	49	52
