# Supplementary material for: Duration of the common cold and similar continuous outcomes should be analyzed on the relative scale: a case study of two zinc lozenge trials
Source: BMC Med Res Methodol. 2017 May 12;17:82. doi: 10.1186/s12874-017-0356-y (PMC5427521; doi:10.1186/s12874-017-0356-y)
Supplement: Supplementary file 3 — Comparison of methods available to calculate the P-values and the 95% CIs for relative effects. (PDF 65 kb) [file 12874_2017_356_MOESM3_ESM.pdf]

Duration of the common cold and similar continuous outcomes should be analyzed on the relative scale: a case study of two zinc lozenge trials

Additional File 3: **Comparison of methods available to calculate the P-values and the 95% CIs for relative effects**

This is additional material to a paper by Hemilä (2017).
https://bmcmedresmethodol.biomedcentral.com

version April 17, 2017

Harri Hemilä
Department of Public Health,
University of Helsinki,
Helsinki, FIN-00014 Finland
harri.hemila@helsinki.fi
http://www.mv.helsinki.fi/home/hemila/
http://www.mv.helsinki.fi/home/hemila/Zinc.htm

Comparison of methods available to calculate the P-values and the 95% CIs for relative effects

**Trial:**	**Mossad (1996) [9]**		**Petrus (1998) [10]**	
**Trial groups:**	Zinc	Placebo	Zinc	Placebo
N:	49	50	52	49
**Mean duration in days:**	5.20	9.20	5.29	7.06
P, variance test to compare the SDs:[1]	$2*10^{-5}$		0.004	
P, Normality test (Shapiro-Wilk):	0.06	0.002	0.003	0.0001
Skewness:	0.55	0.41	0.71	0.86
P(t-test, equal variance):	$1.0*10^{-5}$		0.0080	
P(t-test, unequal variance):	$1.3*10^{-5}$		0.0090	
P(Fisher-Pitman permutation test):	$1.0*10^{-5}$		0.0082	
P(logrank test):	$1.0*10^{-5}$		0.0060	
**The relative scale method used in this study:**				
95% CI:	-62% to -24.9%		-44% to -6.7%	
**Log transformation** of cold duration:				
P, variance test to compare the SDs:	0.9		0.5	
P, Normality test (Shapiro-Wilk):	0.006	0.01	0.03	0.04
Skewness:	-0.74	-0.42	-0.11	-0.02
95% CI:	-56% to -25.1%		-38% to -5.2%	
P (t-test of log durations, equal variance):	$7*10^{-5}$		0.014	
**Taylor series** approach for RoM [5]				
95% CI:	-55% to -29.4%		-39% to -8.2%	
P (Taylor series approach, z-test as in [5]):	$0.04*10^{-5}$		0.0054	
P (Taylor series approach, t-test):	$0.20*10^{-5}$		0.0064	
**Fieller's** approach for RoM (unequal variance)[17]				
95% CI:	-55% to -29.0%		-39% to -7.4%	
**Bootstrap**				
95% CI(mean[Zn] – mean[Placebo=100%]):	-62% to -25.8%		-44% to -7.6%	
95% CI(mean[Zn] / mean[Placebo] – 100%):	-55% to -29.4%		-39% to -8.4%	

The permutation test may be considered as a gold standard since it has no assumptions about the types of distributions of the cold durations. Compared with the permutation test, the t-test of the log transformed data is conservative. Compared with the permutation test, the z-test [5] of the Taylor-series based approach is anticonservative, but the t-test less so. Although there is a significant difference in the variances between the zinc and placebo groups, the t-test on the absolute scale gives a P-value essentially identical with the permutation test and the logrank test.

The relative effect approach used in this study gives 95% CIs that are conservative compared with the 95% CIs calculated with the Taylor-series based approach [5] and the Fieller's approach [17].
