# Supplementary material for: Duration of the common cold and similar continuous outcomes should be analyzed on the relative scale: a case study of two zinc lozenge trials
Source: BMC Med Res Methodol. 2017 May 12;17:82. doi: 10.1186/s12874-017-0356-y (PMC5427521; doi:10.1186/s12874-017-0356-y)
Supplement: Supplementary file 4 — Description of the calculations. (PDF 98 kb) [file 12874_2017_356_MOESM4_ESM.pdf]

Duration of the common cold and similar continuous outcomes should be analyzed on the relative scale: a case study of two zinc lozenge trials

Additional File 4: **Description of the calculations**

This is additional material to a paper by Hemilä (2017).
https://bmcmedresmethodol.biomedcentral.com

version April 17, 2017

Harri Hemilä
Department of Public Health,
University of Helsinki,
Helsinki, FIN-00014 Finland
harri.hemila@helsinki.fi
http://www.mv.helsinki.fi/home/hemila/
http://www.mv.helsinki.fi/home/hemila/Zinc.htm

Page

2 Mossad (1996) calculations

6 Petrus (1998) calculations

10 Taylor series calculation, Mossad (1996) and Petrus (1998)

11 Mossad (1996) Bootstrap for Additional file 3

12 Petrus (1998) Bootstrap for Additional file 3

13 Mossad (1996) Bootstrap for 90% percentile to main text

14 Petrus (1998) Bootstrap for 90% percentile to main text

```
> # MOSSAD
>
> Mossad <- read.csv("~/Mossad_2017_4.csv")

> Mossad$LnDays <- log(Mossad$Days)
> Mossad$RelDays <- Mossad$Days*100/mean(Mossad$Days[Mossad$Zinc == 0]) # Placebo mean = 100%
>
> MossadZn <- subset(Mossad, Zinc == 1)
> MossadPL <- subset(Mossad, Zinc == 0)

>
> # MossadZn
> (mean(MossadZn$Days))
[1] 5.204
> (sd(MossadZn$Days))
[1] 2.828
> (mean(MossadPL$Days))
[1] 9.2
> (sd(MossadPL$Days))
[1] 5.318
>
> var.test(MossadZn$Days,MossadPL$Days)
         F test to compare two variances
data:  MossadZn$Days and MossadPL$Days
F = 0.28, num df = 48, denom df = 49, p-value = 2e-05
alternative hypothesis: true ratio of variances is not equal to 1
95 percent confidence interval:  0.1601 0.5003
sample estimates: ratio of variances  0.2828

> (mean(MossadZn$Days) - mean(MossadPL$Days))
[1] -3.996
>
> MossadAbs <- lm(Mossad$Days ~ Mossad$Zinc )
> summary(MossadAbs)

Coefficients:
               Estimate Std. Error t value Pr(>|t|)
(Intercept)      9.200      0.604   15.23   <2e-16 ***
Mossad$Zinc1    -3.996      0.859   -4.65    1e-05 ***

Residual standard error: 4.27 on 97 degrees of freedom
Multiple R-squared:  0.183,     Adjusted R-squared:  0.174
F-statistic: 21.7 on 1 and 97 DF,  p-value: 1.03e-05

> confint(MossadAbs)
                 2.5 % 97.5 %
(Intercept)   8.001 10.399
Mossad$Zinc1 -5.700 -2.292
>
> quantile(MossadZn$Days, c(.25, .50, .75, .90))
25% 50% 75% 90%
  3   5   7   9
> quantile(MossadPL$Days, c(.25, .50, .75, .90))
25% 50% 75% 90%
  5   8  14  17
>
> (mean(MossadZn$RelDays))  # Relative scale, normalized to placebo mean = 100%
[1] 56.57
> (sd(MossadZn$RelDays))
[1] 30.74
> (mean(MossadPL$RelDays))
[1] 100
> (sd(MossadPL$RelDays))
[1] 57.81
>
```

```
> RoM <-mean(MossadZn$Days)/mean(MossadPL$Days)
> RoM
[1] 0.5657
> PercentageEffect <- 100*(RoM-1)
> PercentageEffect
[1] -43.43
>
> MossadRel <- lm(Mossad$RelDays ~ Mossad$Zinc )
> summary(MossadRel)

Coefficients:
               Estimate Std. Error t value Pr(>|t|)
(Intercept)     100.00       6.57   15.23   <2e-16 ***
Mossad$Zinc1    -43.43       9.33   -4.65    1e-05 ***

Residual standard error: 46.4 on 97 degrees of freedom
Multiple R-squared:  0.183,     Adjusted R-squared:  0.174
F-statistic: 21.7 on 1 and 97 DF,  p-value: 1.03e-05

> confint(MossadRel)
                 2.5 % 97.5 %
(Intercept)   86.97 113.03
Mossad$Zinc1 -61.96 -24.91
>
> ########## Analyses for Additional file 3
>
> var.test(MossadZn$Days,MossadPL$Days)
         F test to compare two variances
data:  MossadZn$Days and MossadPL$Days
F = 0.28, num df = 48, denom df = 49, p-value = 2e-05
alternative hypothesis: true ratio of variances is not equal to 1
95 percent confidence interval:  0.1601 0.5003
sample estimates: ratio of variances 0.2828

>
> shapiro.test(MossadZn$Days)
         Shapiro-Wilk normality test
data:  MossadZn$Days
W = 0.96, p-value = 0.06

> shapiro.test(MossadPL$Days)
         Shapiro-Wilk normality test
data:  MossadPL$Days
W = 0.92, p-value = 0.002

>
> skewness(MossadZn$Days)
[1] 0.555
> skewness(MossadPL$Days)
[1] 0.4152
>
> t.test(Mossad$Days ~ Mossad$Zinc, paired=F, var.equal = T)
         Two Sample t-test
data:  Mossad$Days by Mossad$Zinc
t = 4.654, df = 97, p-value = 1.03e-05
alternative hypothesis: true difference in means is not equal to 0
95 percent confidence interval:  2.29168 5.70016
sample estimates:
mean in group 0 mean in group 1
         9.20000         5.20408


> t.test(Mossad$Days ~ Mossad$Zinc, paired=F, var.equal = F)
         Welch Two Sample t-test
data:  Mossad$Days by Mossad$Zinc
t = 4.68, df = 74.99, p-value = 1.25e-05
alternative hypothesis: true difference in means is not equal to 0
```

```
95 percent confidence interval:  2.29507 5.69677
sample estimates:
mean in group 0 mean in group 1
         9.20000         5.20408

> oneway_test(Mossad$Days ~ Mossad$Zinc, distribution = "exact")
         Exact Two-Sample Fisher-Pitman Permutation Test

data:  Mossad$Days by Mossad$Zinc (0, 1)
Z = 4.229, p-value = 1.04e-05
alternative hypothesis: true mu is not equal to 0

> MossadSurv <- Surv(Mossad$Days, Mossad$Cured)
> survdiff(MossadSurv ~ Mossad$Zinc, rho = 0) # logrank

                  N Observed Expected (O-E)^2/E (O-E)^2/V
Mossad$Zinc=0 50       44     61.3      4.89      19.5
Mossad$Zinc=1 49       47     29.7     10.10      19.5

 Chisq= 19.5  on 1 degrees of freedom, p= 9.92e-06
>
> var.test(MossadZn$LnDays,MossadPL$LnDays)     #  log transformed data
         F test to compare two variances
data:  MossadZn$LnDays and MossadPL$LnDays
F = 0.95, num df = 48, denom df = 49, p-value = 0.9
alternative hypothesis: true ratio of variances is not equal to 1
95 percent confidence interval:  0.5399 1.6868
sample estimates: ratio of variances 0.9535

> shapiro.test(MossadZn$LnDays)
         Shapiro-Wilk normality test
data:  MossadZn$LnDays
W = 0.93, p-value = 0.006

> shapiro.test(MossadPL$LnDays)
         Shapiro-Wilk normality test
data:  MossadPL$LnDays
W = 0.94, p-value = 0.01

> skewness(MossadZn$LnDays)
[1] -0.7419
> skewness(MossadPL$LnDays)
[1] -0.4244
>
> MossadLn <- lm(Mossad$LnDays ~ Mossad$Zinc )
> summary(MossadLn)
Coefficients:
               Estimate Std. Error t value Pr(>|t|)
(Intercept)    2.0249     0.0934   21.67  < 2e-16 ***
Mossad$Zinc1  -0.5525     0.1328   -4.16  6.9e-05 ***

Residual standard error: 0.661 on 97 degrees of freedom
Multiple R-squared:  0.151,     Adjusted R-squared:  0.143
F-statistic: 17.3 on 1 and 97 DF,  p-value: 6.89e-05

> confint(MossadLn)
                  2.5 %  97.5 %
(Intercept)   1.8394  2.2103
Mossad$Zinc1 -0.8161 -0.2889
> (exp(confint(MossadLn))-1)        # 95% CI on the log scale, back-transforming
                  2.5 %  97.5 %
(Intercept)   5.2930  8.1189
Mossad$Zinc1 -0.5578 -0.2509
```

Fieller's approach:

"This function implements the t-test for the ratio of two means and Fiellers confidence interval for the ratio of two means assuming mutually independent Gaussian errors with homogeneous variances, …
With the argument var.equal=FALSE (default), the t-test for the ratio of two means assuming mutually independent Gaussian errors and possibly heterogeneous group variances (Tamhane and Logan, 2004) is implemented."
https://cran.r-project.org/web/packages/mratios/index.html (mratios.pdf)
[17] Tamhane AC, Logan BR. Finding the maximum safe dose level for heteroscedastic data. J Biopharm Stat 2004;14:843-56.
http://dx.doi.org/10.1081/BIP-200035413
https://www.ncbi.nlm.nih.gov/pubmed/15587967

```
> t.test.ratio(MossadZn$Days, MossadPL$Days, data=Mossad, alternative = "two.sided",
+              rho = 1, var.equal = FALSE, conf.level = 0.95)

         Ratio t-test for unequal variances      ## Fieller's approach
data:  x and y
t = -4.7, df = 75, p-value = 1e-05
alternative hypothesis: true ratio of means is not equal to 1
95 percent confidence interval:   0.4518 0.7101
sample estimates:
mean x mean y    x/y
5.2041 9.2000 0.5657

> ( c( 0.4517684, 0.7101461, 0.565661) -1)    # Transformation to percentage scale
[1] -0.5482 -0.2899 -0.4343
>
```

```
> MossadCox   <- coxph(MossadSurv ~ Mossad$Zinc, method = "efron")  # Cox regression
> MossadCox
Call:
coxph(formula = MossadSurv ~ Mossad$Zinc, method = "efron")

                 coef exp(coef) se(coef)   z       p
Mossad$Zinc1 1.016     2.763    0.237 4.3 1.7e-05

Likelihood ratio test=18.9  on 1 df, p=1.36e-05
n= 99, number of events= 91
> exp(confint(MossadCox))
               2.5 % 97.5 %
Mossad$Zinc1 1.738  4.392
```

```
> #  PETRUS
>
> Petrus <- read.csv("~/Petrus_2017_4.csv")

> Petrus$LnDays <- log(Petrus$Days)
> Petrus$RelDays <- Petrus$Days*100/mean(Petrus$Days[Petrus$Zinc == 0]) # Placebo mean = 100%
>
> PetrusZn <- subset(Petrus, Zinc == 1)
> PetrusPL <- subset(Petrus, Zinc == 0)

>
> # PetrusZn
> (mean(PetrusZn$Days))
[1] 5.28846
> (sd(PetrusZn$Days))
[1] 2.56933
> (mean(PetrusPL$Days))
[1] 7.06122
> (sd(PetrusPL$Days))
[1] 3.9073
>
> var.test(PetrusZn$Days,PetrusPL$Days)
         F test to compare two variances
data:  PetrusZn$Days and PetrusPL$Days
F = 0.4324, num df = 51, denom df = 48, p-value = 0.00365
alternative hypothesis: true ratio of variances is not equal to 1
95 percent confidence interval:  0.245433 0.758092
sample estimates: ratio of variances  0.432399


>
> (mean(PetrusZn$Days) - mean(PetrusPL$Days))
[1] -1.77276
>
> PetrusAbs <- lm(Petrus$Days ~ Petrus$Zinc )
> summary(PetrusAbs)

Coefficients:
               Estimate Std. Error t value Pr(>|t|)
(Intercept)     7.061      0.470   15.04   <2e-16 ***
Petrus$Zinc1   -1.773      0.654   -2.71    0.008 **

Residual standard error: 3.29 on 99 degrees of freedom
Multiple R-squared:  0.069,     Adjusted R-squared:  0.0596
F-statistic: 7.34 on 1 and 99 DF,  p-value: 0.00795

> confint(PetrusAbs)
                   2.5 %    97.5 %
(Intercept)   6.12956  7.992894
Petrus$Zinc1 -3.07120 -0.474326
>
> quantile(PetrusZn$Days, c(.25, .50, .75, .90))
25% 50% 75% 90%
  3   5   7   8
> quantile(PetrusPL$Days, c(.25, .50, .75, .90))
25% 50% 75% 90%
  4   6   8  14
>
> (mean(PetrusZn$RelDays))
[1] 74.8944
> (sd(PetrusZn$RelDays))
[1] 36.3864
> (mean(PetrusPL$RelDays))
[1] 100
> (sd(PetrusPL$RelDays))
[1] 55.3346
>
> RoM <-mean(PetrusZn$Days)/mean(PetrusPL$Days)
> RoM
[1] 0.748944
> PercentageEffect <- 100*(RoM-1)
> PercentageEffect
[1] -25.1056
>
```

```
> PetrusRel <- lm(Petrus$RelDays ~ Petrus$Zinc )
> summary(PetrusRel)

Coefficients:
                Estimate Std. Error t value Pr(>|t|)
(Intercept)    100.00       6.65   15.04   <2e-16 ***
Petrus$Zinc1   -25.11       9.27   -2.71    0.008 **

Residual standard error: 46.5 on 99 degrees of freedom
Multiple R-squared:  0.069,     Adjusted R-squared:  0.0596
F-statistic: 7.34 on 1 and 99 DF,  p-value: 0.00795

> confint(PetrusRel)
                  2.5 %    97.5 %
(Intercept)   86.8058 113.19416
Petrus$Zinc1 -43.4939  -6.71734
>
> ########## Analyses for Additional file 3
>
> var.test(PetrusZn$Days,PetrusPL$Days)

         F test to compare two variances

data:  PetrusZn$Days and PetrusPL$Days
F = 0.4324, num df = 51, denom df = 48, p-value = 0.00365
alternative hypothesis: true ratio of variances is not equal to 1
95 percent confidence interval:
 0.245433 0.758092
sample estimates:
ratio of variances
            0.432399

> shapiro.test(PetrusZn$Days)
         Shapiro-Wilk normality test
data:  PetrusZn$Days
W = 0.9234, p-value = 0.00251

> shapiro.test(PetrusPL$Days)
         Shapiro-Wilk normality test
data:  PetrusPL$Days
W = 0.8719, p-value = 7.51e-05

>
> skewness(PetrusZn$Days)
[1] 0.712531
> skewness(PetrusPL$Days)
[1] 0.861827
>
> t.test(Petrus$Days ~ Petrus$Zinc, paired=F, var.equal = T)
         Two Sample t-test
data:  Petrus$Days by Petrus$Zinc
t = 2.709, df = 99, p-value = 0.00795
alternative hypothesis: true difference in means is not equal to 0
95 percent confidence interval:  0.474326 3.071200
sample estimates:
mean in group 0 mean in group 1
         7.06122         5.28846

> t.test(Petrus$Days ~ Petrus$Zinc, paired=F, var.equal = F)
         Welch Two Sample t-test
data:  Petrus$Days by Petrus$Zinc
t = 2.677, df = 82.23, p-value = 0.00896
alternative hypothesis: true difference in means is not equal to 0
95 percent confidence interval:  0.455472 3.090053
sample estimates:
mean in group 0 mean in group 1
         7.06122         5.28846

>
> oneway_test(Petrus$Days ~ Petrus$Zinc, distribution = "exact")
         Exact Two-Sample Fisher-Pitman Permutation Test
data:  Petrus$Days by Petrus$Zinc (0, 1)
Z = 2.627, p-value = 0.00815
alternative hypothesis: true mu is not equal to 0
```

```
> PetrusSurv <- Surv(Petrus$Days, Petrus$Cured)
> survdiff(PetrusSurv ~ Petrus$Zinc, rho = 0) # logrank
Call:
survdiff(formula = PetrusSurv ~ Petrus$Zinc, rho = 0)

                  N Observed Expected (O-E)^2/E (O-E)^2/V
Petrus$Zinc=0 49       49     60.6      2.21      7.56
Petrus$Zinc=1 52       52     40.4      3.31      7.56

 Chisq= 7.6  on 1 degrees of freedom, p= 0.00597
>
> var.test(PetrusZn$LnDays,PetrusPL$LnDays)     # log transformed data
         F test to compare two variances
data:  PetrusZn$LnDays and PetrusPL$LnDays
F = 0.8206, num df = 51, denom df = 48, p-value = 0.487
alternative hypothesis: true ratio of variances is not equal to 1
95 percent confidence interval:  0.465804 1.438773
sample estimates: ratio of variances  0.820644

>
> shapiro.test(PetrusZn$LnDays)
         Shapiro-Wilk normality test
data:  PetrusZn$LnDays
W = 0.9497, p-value = 0.0284

> shapiro.test(PetrusPL$LnDays)
         Shapiro-Wilk normality test
data:  PetrusPL$LnDays
W = 0.9516, p-value = 0.0428

>
> skewness(PetrusZn$LnDays)
[1] -0.107644
> skewness(PetrusPL$LnDays)
[1] -0.0151264
>
> PetrusLn <- lm(Petrus$LnDays ~ Petrus$Zinc )
> summary(PetrusLn)

Coefficients:
               Estimate Std. Error t value Pr(>|t|)
(Intercept)    1.8083     0.0751   24.08   <2e-16 ***
Petrus$Zinc1  -0.2611     0.1047   -2.49    0.014 *

Residual standard error: 0.526 on 99 degrees of freedom
Multiple R-squared:  0.0591,    Adjusted R-squared:  0.0496
F-statistic: 6.22 on 1 and 99 DF,  p-value: 0.0143

> confint(PetrusLn)
                   2.5 %     97.5 %
(Intercept)   1.659256  1.9573086
Petrus$Zinc1 -0.468766 -0.0533796
> (exp(confint(PetrusLn))-1)          # 95% CI on the log scale, back-transforming
                   2.5 %     97.5 %
(Intercept)   4.255399  6.0802457
Petrus$Zinc1 -0.374226 -0.0519799
```

```
> t.test.ratio(PetrusZn$Days, PetrusPL$Days, data=Petrus, alternative = "two.sided",
+               rho = 1, var.equal = FALSE, conf.level = 0.95)

         Ratio t-test for unequal variances      ## Fieller's approach

data:  x and y
t = -2.677, df = 82.23, p-value = 0.00896
alternative hypothesis: true ratio of means is not equal to 1
95 percent confidence interval:  0.610357 0.925347
sample estimates:
  mean x   mean y      x/y
5.288462 7.061224 0.748944

> ( c(  0.6103566, 0.9253466, 0.748944) -1)   # Transformation to percentage scale
[1] -0.3896434 -0.0746534 -0.2510560
>
```

```
> PetrusCox   <- coxph(PetrusSurv ~ Petrus$Zinc, method = "efron") # Cox regression
> PetrusCox
Call:
coxph(formula = PetrusSurv ~ Petrus$Zinc, method = "efron")


                 coef exp(coef) se(coef)    z      p
Petrus$Zinc1 0.573     1.774    0.214 2.68 0.0073

Likelihood ratio test=7.3  on 1 df, p=0.00691
n= 101, number of events= 101
> exp(confint(PetrusCox))
                  2.5 %  97.5 %
Petrus$Zinc1 1.16717 2.69598
```

```
> #  Taylor series calculations for 95% CI
> # Mossad
>
> (N1M =length(MossadZn$Days))
[1] 49
> (M1M =mean(MossadZn$Days))
[1] 5.20408
> (SD1M =sd(MossadZn$Days))
[1] 2.82828
>
> (N2M = length(MossadPL$Days))
[1] 50
> (M2M = mean(MossadPL$Days))
[1] 9.2
> (SD2M =sd(MossadPL$Days))
[1] 5.31843
>
> MZN <- (SD1M/M1M)**2/N1M # [SD(Zn)/Mean(Zn)]**2 /N(Zn)
> MPL <- (SD2M/M2M)**2/N2M # [SD(Pl)/Mean(Pl)]**2 /N(Pl)
> (Mln_SEpool <- sqrt(MZN + MPL))
[1] 0.112746
> Mln_RoM      <- log(M1M/M2M)
> (Mln_LowCI   <- Mln_RoM - 1.96*Mln_SEpool)
[1] -0.790742
> (Mln_HiCI    <- Mln_RoM + 1.96*Mln_SEpool)
[1] -0.348779
> (MLowCI_rel <- exp(Mln_LowCI))
[1] 0.453508
> (MHiCI_rel  <- exp(Mln_HiCI))
[1] 0.705549
> (MLowCI      <- (MLowCI_rel-1)*100)  # to the percentage effect scale
[1] -54.6492
> (MHiCI       <- (MHiCI_rel-1)*100)   # to the percentage effect scale
[1] -29.4451
> (M_Z = Mln_RoM/Mln_SEpool)
[1] -5.0535
> (2*pnorm(M_Z))      # z-test as in [5]
[1] 4.33784e-07
> (2*pt(M_Z,N1M+N2M-2)) # t-test
[1] 2.04158e-06
>
> # Petrus
>
> (N1P =length(PetrusZn$Days))
[1] 52
> (M1P =mean(PetrusZn$Days))
[1] 5.28846
> (SD1P =sd(PetrusZn$Days))
[1] 2.56933
>
> (N2P = length(PetrusPL$Days))
[1] 49
> (M2P = mean(PetrusPL$Days))
[1] 7.06122
> (SD2P =sd(PetrusPL$Days))
[1] 3.9073
>
> PZN <- (SD1P/M1P)**2/N1P # [SD(Zn)/Mean(Zn)]**2 /N(Zn)
> PPL <- (SD2P/M2P)**2/N2P # [SD(Pl)/Mean(Pl)]**2 /N(Pl)
> (Pln_SEpool <- sqrt(PZN + PPL))
[1] 0.103865
> Pln_RoM      <- log(M1P/M2P)
> (Pln_LowCI   <- Pln_RoM - 1.96*Pln_SEpool)
[1] -0.492667
> (Pln_HiCI    <- Pln_RoM + 1.96*Pln_SEpool)
[1] -0.0855153
> (PLowCI_rel <- exp(Pln_LowCI))
[1] 0.610995
> (PHiCI_rel  <- exp(Pln_HiCI))
[1] 0.918039
> (PLowCI      <- (PLowCI_rel-1)*100)  # to percentage scale
[1] -38.9005
> (PHiCI       <- (PHiCI_rel-1)*100)   # to percentage scale
[1] -8.19609
> (P_Z = Pln_RoM/Pln_SEpool)
[1] -2.78333
> (2*pnorm(P_Z))      # z-test
[1] 0.00538042
> (2*pt(P_Z,N1P+N2P-2)) # t-test
[1] 0.0064452
```

```
#  BOOTSTRAP

# Ratio of means

RatioM <- function(Mossad, d){
  EMR=Mossad[d,]
  return(100*(mean(EMR$RelDays[EMR$Zinc == 1])/mean(EMR$RelDays[EMR$Zinc == 0]) -1))
}

bM<-boot(Mossad, RatioM, strata = Mossad$Zinc, R = 100000)
(ci = boot.ci(bM, type =c("basic","bca")))

#
BOOTSTRAP CONFIDENCE INTERVAL CALCULATIONS
Based on 100000 bootstrap replicates

CALL :
  boot.ci(boot.out = bM, type = c("basic", "bca"))

Intervals :
  Level      Basic                BCa
95%   (-57.38, -32.38 )   (-54.45, -29.40 )
Calculations and Intervals on Original Scale

###
# Difference in means

DiffM <- function(Mossad, d){
  EMD=Mossad[d,]
  return(mean(EMD$RelDays[EMD$Zinc == 1]) -mean(EMD$RelDays[EMD$Zinc == 0]))
}

bMD<-boot(Mossad, DiffM, R = 100000)
(ci = boot.ci(bMD, type =c("basic","bca")))


#
BOOTSTRAP CONFIDENCE INTERVAL CALCULATIONS
Based on 100000 bootstrap replicates

CALL :
  boot.ci(boot.out = bMD, type = c("basic", "bca"))

Intervals :
  Level      Basic                BCa
95%   (-61.40, -25.13 )   (-62.06, -25.80 )
Calculations and Intervals on Original Scale
```

```
RatioP <- function(Petrus, d){
  EPR=Petrus[d,]
  return(100*(mean(EPR$RelDays[EPR$Zinc == 1])/mean(EPR$RelDays[EPR$Zinc == 0]) -1))
}

bPR<-boot(Petrus, RatioP, strata = Petrus$Zinc, R = 100000)
(ci = boot.ci(bPR, type =c("basic","bca")))

#
BOOTSTRAP CONFIDENCE INTERVAL CALCULATIONS
Based on 100000 bootstrap replicates

CALL :
  boot.ci(boot.out = bPR, type = c("basic", "bca"))

Intervals :
  Level      Basic                BCa
95%   (-42.05, -11.58 )   (-38.85,  -8.45 )
Calculations and Intervals on Original Scale



###
# Difference in means

DiffP <- function(Petrus, d){
  EPD=Petrus[d,]
  return(mean(EPD$RelDays[EPD$Zinc == 1]) - mean(EPD$RelDays[EPD$Zinc == 0]))
}

bPD<-boot(Petrus, DiffP, R = 100000)
(ci = boot.ci(bPD, type =c("basic","bca")))

#
BOOTSTRAP CONFIDENCE INTERVAL CALCULATIONS
Based on 100000 bootstrap replicates

CALL :
  boot.ci(boot.out = bPD, type = c("basic", "bca"))

Intervals :
  Level      Basic                BCa
95%   (-43.16,  -6.57 )   (-44.20,  -7.59 )
Calculations and Intervals on Original Scale
```

```
# 90-percentile bootstraps

Quant90DiffM <- function(Mossad, d){
  EPD=Mossad[d,]
  return(quantile(EPD$Days[EPD$Zinc == 1], .9) - quantile(EPD$Days[EPD$Zinc == 0],0.9))
}

bMq90<-boot(Mossad, Quant90DiffM, R = 50000)
(ci = boot.ci(bMq90, type =c("basic","bca")))

#
BOOTSTRAP CONFIDENCE INTERVAL CALCULATIONS
Based on 50000 bootstrap replicates

CALL :
  boot.ci(boot.out = bMq90, type = c("basic", "bca"))

Intervals :
  Level      Basic              BCa
95%   (-11.0,  -5.0 )   (-11.0,  -5.6 )
Calculations and Intervals on Original Scale

##

Quant90RatioM <- function(Mossad, d){
  EPD=Mossad[d,]
  return(quantile(EPD$Days[EPD$Zinc == 1], .9)/quantile(EPD$Days[EPD$Zinc == 0],0.9) -1)
}

bMq90<-boot(Mossad, Quant90RatioM, R = 50000)
(ci = boot.ci(bMq90, type =c("basic","bca")))

#
BOOTSTRAP CONFIDENCE INTERVAL CALCULATIONS
Based on 50000 bootstrap replicates

CALL :
  boot.ci(boot.out = bMq90, type = c("basic", "bca"))

Intervals :
  Level      Basic              BCa
95%   (-0.6250, -0.3529 )   (-0.5789, -0.3125 )
Calculations and Intervals on Original Scale
```

```
Quant90DiffP <- function(Petrus, d){
  EPD=Petrus[d,]
  return(quantile(EPD$Days[EPD$Zinc == 1], .9) - quantile(EPD$Days[EPD$Zinc == 0],0.9))
}

bPq90<-boot(Petrus, Quant90DiffP, R = 50000)
(ci = boot.ci(bPq90, type =c("basic","bca")))

#
BOOTSTRAP CONFIDENCE INTERVAL CALCULATIONS
Based on 50000 bootstrap replicates

CALL :
  boot.ci(boot.out = bPq90, type = c("basic", "bca"))

Intervals :
  Level      Basic                BCa
95%   (-10.7,  -5.0 )   ( -8.0,  -4.5 )
Calculations and Intervals on Original Scale
Some BCa intervals may be unstable

###

Quant90RatioP <- function(Petrus, d){
  EPD=Petrus[d,]
  return(quantile(EPD$Days[EPD$Zinc == 1], .9)/quantile(EPD$Days[EPD$Zinc == 0],0.9) -1)
}

bPq90<-boot(Petrus, Quant90RatioP, R = 50000)
(ci = boot.ci(bPq90, type =c("basic","bca")))

#
BOOTSTRAP CONFIDENCE INTERVAL CALCULATIONS
Based on 50000 bootstrap replicates

CALL :
  boot.ci(boot.out = bPq90, type = c("basic", "bca"))

Intervals :
  Level      Basic                BCa
95%   (-0.7441, -0.3638 )   (-0.5340, -0.3333 )
Calculations and Intervals on Original Scale
```
